# Subcellular Expression of Maspin in Colorectal Cancer: Friend or Foe

**DOI:** 10.3390/cancers13030366

**Published:** 2021-01-20

**Authors:** Simona Gurzu, Ioan Jung

**Affiliations:** Department of Pathology, George Emil Palade University of Medicine, Pharmacy, Sciences and Technology, 540139 Targu-Mures, Romania; janos.jung@umfst.ro

**Keywords:** maspin, colorectal, *Saccharomyces cerevisiae*, epithelial mesenchymal transition, Wnt pathway, inflammatory bowel disease, immunohistochemistry

## Abstract

**Simple Summary:**

In this article we have highlighted the possible role of nuclear maspin in identification of tumor cells “on the point of budding” and the epithelial mesenchymal transition phenomenon of these cells, along with a deep exploration of the maspin-molecular mediated mechanisms in colorectal cancer.

**Abstract:**

In this review the authors aimed to emphasize the practical value of nuclear expression of the mammary serine protease inhibitor (maspin), also known as serpin B5 protein, in colorectal carcinoma (CRC), from pre-malignant disorders to carcinogenesis and metastasis. As the role of maspin is controversial and not yet understood, the present update highlights the latest data revealed by literature which were filtrated through the daily experience of the authors, which was gained at microscopic examination of maspin expression in CRCs and other tumors for daily diagnosis. Data regarding the subcellular localization of maspin, in correlation with the microsatellite status, grade of tumor dedifferentiation, and epithelial-mesenchymal transition (EMT) phenomenon of the tumor buds were presented with details. An original observation refers to the maspin capacity to mark the tumor cells which are “at the point of budding” that were previously considered as having “hybrid EMT phenotype”. It refers to the transitional status of tumor cell that is between “epithelial status” and “mesenchymal status”. The second original hypothesis highlights the possible role of maspin in dysregulating the intestinal microbiota, in patients with idiopathic inflammatory bowel diseases (IBD) and inducing IBD-related CRC. The dynamic process of budding and EMT of tumor buds, possible mediated by maspin, needs further investigation and validation in many human CRC samples. The histological and molecular data reveal that synthesis of maspin-based therapeutics might represent a novel individualized therapeutic strategy for patients with CRC.

## 1. Introduction

Personalized health care in cancer tends to be based on histological and molecular classification of solid cancers and should be realized using standardized and reproducible biomarkers [1]. In colorectal carcinomas (CRCs), tumor budding degree should be mentioned in the histopathological reports as an indicator of local aggressivity and metastatic risk [1,2].

Although the budding degree can be estimated on hematoxylin and eosin (HE) slides supplementary immunohistochemical (IHC) stains are recommended to be performed, for a proper estimation of the tumor buds in the invasive area [1,2,3]. In most of the cases, cytokeratins (CK) are used for quantification but the reported inter-observer variability is high [1,2].

The third aspect refers to the cytoskeletal arrangement of the tumor cells, especially in the buds, with occurrence of the epithelial mesenchymal transition (EMT) phenomenon. EMT is characterized by loss of adhesion markers such E-cadherin, loss of membrane positivity, or nuclear translocation for ß-catenin and, in some cases, acquirement of positivity for vimentin, Slug, Twist, or other mesenchymal markers [1,2]. 

In the present review, the authors intended to present a critical review of data published in English literature, which emphasized the role of the mammary serine protease inhibitor (maspin) in CRC. Upon checking the MedLine and Web of Science databases, fewer than 50 papers were found to show data about maspin expression in CRCs, all of them being published between 2002 and 2020. We chose, for the present update, those few papers which were focused on subcellular expression of maspin (nuclear vs. cytoplasm) in CRC and aimed to present the possible role of maspin as a link between tumor budding degree and EMT of tumor cells. For a proper understanding of maspin, data about its expression in other tumors and its molecular properties were also used. 

## 2. General Data about Maspin

Being also known as the serpin B5 or peptidase inhibitor 5, maspin is an unsusual non-inhibitory member of the the serine protease inhibitor (serpin) superfamily [4,5]. Maspin gene is located to chromosome 18q21.3 of exon 2 and was firstly described by Zou et al. in 1994 [5,6,7]. 

Maspin is a 42 kDa ovalbumin-like soluble non-glycosylated or phosphorylated protein which containes three β-sheets (A, B, C), eight cysteine residues and nine alphahelices (A–I) [7,8]. It has a flexible short hydrophobic reactive center loop/site loop, responsible for cell binding and cell adhesion, and does not possess a hydrophobic amino terminus [8,9]. The chemical structure of maspin is similar to those of α1-antitrypsin but also with the neutrophil-monocyte elastase inhibitors [5,8,9].

Maspin was firstly isolated from human mammary epithelial cells [5,6,7] and then from *Saccharomyces cerevisiae* [8]. A review published in 2019 by Banias et al. showed that, in normal organs and tissues, maspin can be present in epithelial and non-epithelial structures [6]. It can mark urothelium, squamous epithelium, basal cells of the prostate and bronchial epithelium, placental cyto- and syncytio-trophoblasts, fibroblasts, myoepithelial cells of the mammary gland, endometrium, mucosa of the gastrointestinal tract (esophagus, stomach, small intestine, colon and rectum), testis, thymus, as well as corneal keratocytes, epithelial and endothelial cells [4,5,7,9,10,11,12]. Any human tissue might express maspin, mainly in the cytoplasm, secretory vesicles and cell membrane but the expression level is not similar [4,9]. 

Maspin acts as a pro-apoptotic protein with role in implantation of the embryos into the uterine wall, embryonic development, cell adhesion, and oxidative stress response [7,9,13]. It can also inhibit the urokinase plasminogen activator (uPA) and tissue plasminogen activator and modulate the mammary gland morphogenesis during pregnancy; high maspin expression was associated with low milk production [7]. Developing the human placenta, maintaining activity of extravillous trophoblast cells and regulation of their migration and invasion seems to also be done by maspin via modulation of some pro-angiogenic/pro-lymphangiogenic markers such Vascular Endothelial Growth Factor C (VEGF-C) and its receptors (VEGFR-2 and VEGFR-3) [7,14]. 

In non-tumor pathologies, maspin is involved in wound healing [7] but was also described as a co-pathogenetic factor of autoimmune disorders [15]. In psoriasis, maspin is overexpressed in the hyperplastic epidermis [15].

## 3. Maspin Expression in Malignant Tumors

In malignancies, maspin acts as a tumor supressor gene that inhibit invasion, angiogenesis and endothelial cell migration towards basic fibroblast growth factor and regulates apoptosis [6,10,11]. It can also have oncogenic activity and its role depends on the tumor histology and tumor localization [9]. Besides the tissue-specificity, the role of maspin gene also depends on the subcellular localization of the maspin protein [2,6,11,12]. Because sodium peroxidovanadate, a tyrosine phosphatase inhibitor, induced experimentally upregulation of cytoplasmic maspin, it is presumed that subcellular localization is probably regulated by maspin tyrosine phosphorylation [11]. Other authors consider that maspin translocation, from cytoplasm to nucleus, is mediated by mitochondrial phospholipids such cardiolipin [13].

Maspin can be down- or upregulated in tumors but the significance of these changes, correlated with maspin subcellular localization, compared with parental tissues, is far to be understood. Most of the published papers refers to maspin expression in carcinomas [4,6,16,17]. In the previously published reports, maspin was examined in particular in breast carcinomas and CRCs, but it was reported to also mark carcinomas of the oral cavity, esophagus, stomach, larynx, lung, pancreas, thyroid, prostate, ovary and urinary bladder [5,6,17,18,19]. 

Although unusual, maspin was reported to also mark soft tissue sarcomas [18] and melanomas [7,19]. A sun-activated maspin-induced DNA damage was hypothesized to be the pathogenetic mechanism of skin melanoma but the prognostic significance is still unknown [19].

Maspin is a p53-target gene which also depends on the microsatellite status of the tumor cells [1,15,19,20]. In gastric carcinomas, we proved that loss of maspin might be induced by TP53 gene mutations in exon 7 whereas wild-type p53 was hypothesized to be responsible by restoration of the nuclear maspin expression and further decreasing of the metastatic potential [20]. 

In CRC, maspin cytoplasmic positivity is mostly associated with negativity for p53 protein, whereas nuclear positive cases expressed p53 in over 50% of tumor cells [20,21,22]. As maspin can be co-expressed in the cytoplasm or on the cell membrane of the tumor cells, with carcinoembryonic antigen (CEA), it is considered a CEA-interacting biomarker [9]. The serum level of maspin is also correlated with the CEA level, being postulated that a high level of maspin, in the blood of patients with CRC, is an indicator of aggressivity [9].

Being a regulator of apoptosis, maspin is modulated by B-cell lymphoma 2 (Bcl-2) family genes and can metallop with bcl-2 and bax proteins [9,16]. In a pro-apoptotic medium, maspin is translocated from the cytosol to inner mitochondria membrane and induces membrane disruption with further apoptosis [14]. Interaction with other proteins such collagen I and III, glutathione S-transferase, VEGF, early growth response protein 1 (EGR1), p63, interferon regulatory factor 6, γ-linolenic acid, matrix metalloproteinases (MMP) such MMP-2 and β-catenin was also proved [7,9,23]. Interaction with fatty acids such omega-6 EFAs arachidonic acid and α-linolenic acid was denied [7]. 

In tumors such breast cancer maspin level was described to be dependent on the estrogen receptors α and β [7,17]. In line with these data, tamoxifen, which is used as a variant of hormonal therapy, proved to stimulate the secretion of maspin in the myoepithelial cells, without significant changes at mRNA level [7].

**Figure 1:**
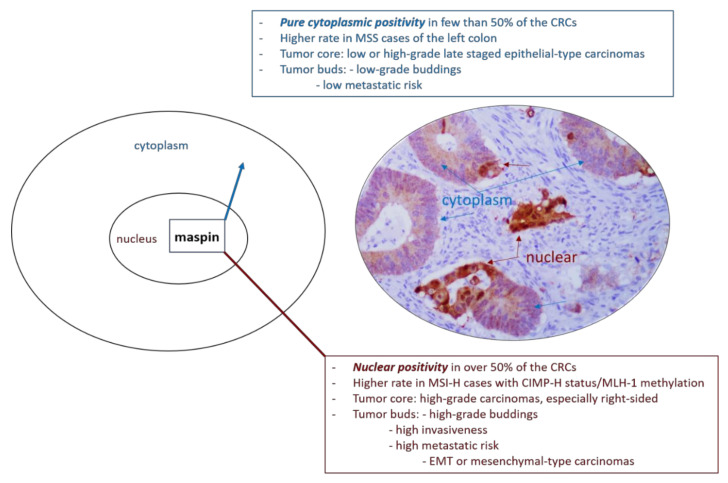
Mechanistic role of subcellular expression of maspin in colorectal cancer.

Regarding gastrointestinal cancers, in both gastric carcinomas and CRC, cytoplasmic maspin positivity is considered as an indicator of low metastatic risk (Figure 1) and late recurrence but nuclear positivity is correlated with early recurrence after surgery, especially for advanced stage carcinomas [11,16,24,25,26]. In early stages, nuclear maspin might be related with risk for lymph node metastases [27]. Decreased maspin increases the risk of tumor progression and occurrence of distant metastases [11,12,16,24,25]. In CRC maspin is even described as an immunogen or “autoantibody-inducing autoantigen” with immunomodulatory role [4,28,29]. The independent prognostic role of maspin is, however, not accepted by all of the authors [1,26]. 

Due to the few publications about maspin, there are also controversial data regarding the evaluation of the IHC expression of maspin protein. For the immunostains, the subcellular localization is indicated to be quantified based on the intensity, percentage and localization in the tumor cells [16,20]. Using a cut-off of 10–25%, cases can be grouped in: negative cases, carcinomas with cytoplasm predominance (cytoplasmic high and nuclear low), nuclear expression (cytoplasmic low and nuclear high), respectively with mixed expression (dual positivity, with high cytoplasm and high nuclear intensity) [16,20]. In daily practice, nuclear expression without cytoplasmic positivity is extremely rare [9].

## 4. Maspin and CRC Budding Degree 

The newest international guidelines indicate to consider the tumor budding degree as an independent prognostic factor of CRC [11]. Tumor buds are defined as released “single cancer cells or small poorly differentiated clusters composed of no more than five tumor cells, without gland formation” in the tumor stroma at the tumor invasive front [10,27,30,31]. A consensus was established in 2016 to report CRCs as presenting low or G1- (0–4 buds), moderate or G2- (5–9 buds), and high or G3- (≥10 buds) budding degree [1,2,31]. However, in routine practice, buddings are reported by pathologists as G1, G2, or G3, whereas other histopathological reports classify cases as having low (G1) or high budding intensity (G2 + 3) [11]. In other papers, only cases with more than 9 foci were classified as high-grade CRCs [27]. Although it is recommended to quantify the budding intensity in the invasive front, intratumorally stroma should also be examined, and one high power field (20-fold magnification; one-fold of 0.385 mm^2^) is accepted as sufficient for budding quantification [1,11,27]. These aspects demonstrate the suboptimal interobserver agreement and lack of reproducibility [31].

Some pathologists quantify the buddings based on HE stained slides, whereas others use CKAE1/AE3, CK20, or CK8-18 [1,2,27,30,31], using the rule of a visible presence of the nucleus [31]. However, CK20 can be downregulated or even lost in the poorly differentiated cell clusters which show, in CRCs, neuroendocrine differentiation, EMT or microsatellite instability (MSI), particularly if they are BRAF mutated [3,32,33]. These data emphasize that, although the CKs remain the major cytoskeletal components of the gastrointestinal columnar epithelium, they can be downregulated or even negative in the tumor buds which show disintegration of the actin cytoskeleton, dysregulation of cell junctions or neuroendocrine phenotype [33]. In such cases, CKs might be difficult to be used for quantification of tumor budding degree.

As Lugli et al. mentioned in their recent article [1], an IHC marker that might be used for budding quantification, at least for gastrointestinal carcinomas, is the maspin protein [1,11]. It was found to be easily reproducible but compared with CKs, which are expressed in the cell membrane only, maspin expression can be observed in nuclei and/or cytoplasm [28]. Maspin shows a good concordance with the CKs cocktail (83%; κ = 0.66–0.68) and simplifies identification of the isolated cells and tumor clusters, without marking the apoptotic bodies [9,27]. 

Maspin nuclear expression could indicate a higher grade of dedifferentiation, especially in the invasive front, particularly for high-budding degree CRCs with microsatellite stable status (MSS) [11,28,34,35,36]. It is expressed from early carcinogenesis up to advanced stage carcinomas with unresectable metastases [28,34]. 

Another aspect that must be added is the “dynamic process of budding” [1]. As senior pathologists, we have examined, for our daily diagnosis, over 350 cases of CRCs, from 2010 up to date, and quantified the maspin subcellular expression in these cases. The first results of our team were published in 2013, and we proposed a system of quantification of maspin subcellular expression, which was further certified in other papers elaborated with our Ph.D. students [2,6]. We wish to highlight, in the present article, an unpublished personal observation that refers to the capacity of maspin protein to mark not only the dyscohesive cells from the invasive front, referred to as tumor buds [1,2,6], but also the nuclei of those tumor cells, from the tumor-stroma interaction line, which are “at the point of budding” (Figure 1). As Lugli et al. mentioned, in early stage MSS-CRCs, tumor budding degree is an indicator of the risk of lymph node metastases and informs about the need for radical surgery; budding intensity assists in identifying patients with high-risk stage II CRC that requires adjuvant therapy [1,31]. Identification of the cells “at the point of budding” might help pathologists in identification of such cases. As CKs are expressed in membranes only, they can only show non-specific focal membrane expression in the cells “at the point of budding” whereas poor visibility of the nuclei in the tumor buds might lead to misclassification [31]. If these data are proved on large cohorts, maspin might become the gold standard IHC antibody for budding quantification.

## 5. Maspin and EMT Phenomena

EMT is characterized by loss of the epithelial hallmarks conferred by the adhesion markers such CKs, E-cadherin and ß-catenin and, acquirement of mesenchymal properties which are reflected by loss of membrane positivity for E-cadherin and loss or nuclear translocation for ß-catenin. The epithelial cells showing EMT present a mesenchymal phenotype which is proved by positivity for vimentin, Slug, Twist, ZEB, N-cadherin, fibronectin, matrix metalloproteinases and so on [1,2,30,33]. 

EMT is involved in embryogenesis, tissue repair and tumorigenesis [2]. All the three processes proved to be partially modulated by maspin [7,9,13] but we did not find any study to link maspin by the non-tumor-related EMT phenomenon. 

In CRC, EMT mainly occurs via Wnt/β-catenin pathway which might interact with proteins such a mena (mammalian Ena homolog), arylsulfatase B (ARSB) or maspin [36,37,38]. ARSB is directly correlated with maspin IHC expression, as an indicator of high invasive potential [37]. On the other hand, ARSB gene can inhibit the non-canonical Wnt signaling pathway and subsequently the EMT phenomenon via chondroitin 4-sulfate [36]. In line to these data, we previously proved that a low circulating level of *ARSB* gene (<0.5) is associated with double ARSB/maspin positivity of tumor cells and high grade of tumor budding [37], probably as an indicator of EMT [36,37].

It is theorized but not yet proven that, in CRC, “tumor budding is the histological reflection of the EMT” that might present a specific gene signature [30]. Based on this hypothesis and the positivity or negativity of tumor cells for the EMT-related markers, in the central tumor core (bulk) vs. buds, CRCs can be classified as epithelial- (≥45% of cases) or mesenchymal subtype (<15%) [2], the latest one having a more unfavorable prognosis [1,2]. 

An enigmatic phenomenon is the transitional state of the tumor cell, referred to as “the hybrid EMT phenotype” or “shift from epithelial to mesenchymal molecular profile” [2,30]. During this step, the tumor cells even express both epithelial and mesenchymal markers [1] or present an “epithelial-type core” and “mesenchymal-type buds” [2,30]. The behavior of the “hybrid cases”, which represent approximately 40% of CRCs [2], tends to be more like the mesenchymal-type CRCs than to the epithelial molecular subtype. Because it represents a significant number of cases, this group of carcinomas should be more attentively evaluated in large cohorts. 

The significance of the subcellular localization of maspin is not yet understood. It was recently demonstrated that maspin shows a predominant cytoplasmic positivity in “epithelial CRCs” and the presence of nuclear staining might be an indicator of mesenchymal or hybrid CRCs [2]. In hybrid CRCs, maspin cytoplasmic staining is mainly seen in the tumor core, whereas nuclear predominance can be observed in the tumor buds [2,11]. Nuclear maspin is mainly associated with membrane-to-nuclear translocation of β-catenin, especially in buds [2]. 

As maspin is more often expressed in the invasive front [35,39,40] and its nuclear expression indicate a high-budding degree [27,40], it might be considered an indicator of the EMT phenomenon of the buds and of highly aggressive MSS cases with invasive potential [11,37]. This hypothesis, which was not emphasized by other authors, deserves a deeper investigation. 

## 6. Maspin and Molecular Pathways of Colorectal Carcinogenesis

### 6.1. Maspin and Microsatellite Status

In MSS sporadic colorectal adenocarcinomas, maspin nuclear expression is considered an indicator of high aggressivity, high budding degree but also high grade of de-differentiation [2,11,35]. No maspin nuclear positivity was proved for other histological subtypes such: the MSS neuroendocrine tumors (NET), neuroendocrine carcinomas (NEC), mixed adenoneuroendocrine carcinomas (MANEC) or primary choriocarcinoma of the gastrointestinal tract [41,42,43]. Other neuroendocrine tumors such Merkel cell carcinoma developed in sun-exposed areas or pancreatic mucinous cystic neoplasms with neuroendocrine differentiation proved to show infrequent positivity for maspin [44,45]. 

In MSI-H colorectal adenocarcinomas and MSI-H cell lines (SW48, LOVO, HCT116), maspin expression proved to be upregulated, especially in the cytoplasm, compared with normal colorectal mucosa, and even more upregulated than in MSS cases [22,35]. Because the immune escape mechanisms of tumor buds are different in MSI cases [1], the budding degree is not as helpful in these carcinomas as it is for MSS carcinomas. Maspin upregulation in the tumor core of early stage MSI-H carcinomas seems to be rather an indicator for a longer overall and disease-free survival [4]. Some authors admit a nuclear overexpression [35] while others showed no significant association of subcellular expression with evolution or prognosis of MSI-H cases [4]. 

Kim et al. considered nuclear positivity, in MSI-H adenocarcinomas, an indicator of aggressivity which is correlated with lympho-vascular/perineural invasion, advanced AJCC/UICC tumor stage and CpG island methylator phenotype (CIMP-H status) [46]. 

### 6.2. Maspin and the Serrated Pathway

In patients with CRCs with MSI-H status and BRAF mutations, positivity for CK7 and/or downregulated and even loss of CK20 might be indicators of diagnosis of a serrated pathway CRC, even in cases which do not show the specific morphological features [3,47]. These features are especially specific for tumors of the proximal colon [47]. 

In line with these data and the fact that maspin is upregulated in MSI-H cases [4,35], Rubio et al. showed maspin positivity of serrated polyps and sessile serrated lesions without dysplasia and hypothesized that the serrated pathway of colorectal carcinogenesis might be mediated by maspin [48,49]. They claim that the cytoplasmic expression of maspin might be an indicator of serrated pathway [47]. On the other hand, Kim et al. claimed that nuclear maspin is molecularly associated with CIMP-H rather than MSI-H [46].

### 6.3. Maspin and Colorectal Carcinogenesis via Idiopathic Inflammatory Bowel Disease (IBD)

Aberrant expression of maspin was described in specimens provided from patients with IBD, respectively ulcerative colitis and Crohn’s disease [48,49,50]. Its expression was correlated with the IBD activity and the grade of IBD-induced dysplasia, being overexpressed in active IBD, in both cytoplasm and nuclei, with nuclear predominance in cases with dysplasia [50]. Based on these aspects, Cao et al. induced the supposition of maspin involvement in the genesis of IBD-related CRC, as a molecule which might mark the borderline of the three processes: chronic inflammation, dysplasia and neoplasia [50]. We did not find other studies that confirm or infirm this hypothesis.

As maspin was isolated from *Saccharomyces cerevisiae* [8] and antibodies against *Saccharomyces cerevisiae* (ASCA) can be detected in patients with Crohn’s disease or other autoimmune disorders of the gastrointestinal tract [51,52], it might be supposed that, in patients with IBD and increase IgM and IgG ASCA, the dysregulated intestinal microbiota can occur via cytoplasm-to-nuclear translocation of the maspin protein. Saccharomyces cerevisiae is used in alcoholic and baking industry but is also a component of vaccines [51]. 

We did not identify studies to prove this hypothesis. For this reason, we cannot know if maspin-mediated inflammation-dysplasia-neoplasia process depends on the immunogenic properties of maspin [28,29], the p53-maspin interraction [16,20,53], the molecular mimicry of *Saccharomyces cerevisiae* [51] or it is about the superposed role of the environmental stimuli in dysregulating gut microbiota and inducing IBD. To our best knowledge, no data about the maspin influence upon gut microbiome were published yet.

## 7. Predictive Value of the Subcellular Maspin Expression

### 7.1. Maspin and 5-Fluorouracil (5-FU)

Based on the role of maspin in the regulation of the p53 gene [54,55,56] it was hypothesized that simultaneous maspin/p53 nuclear positivity is an indicator of response to 5-FU based therapy [26,28,54,55]. On the other hand, maspin negative/p53 positivity can indicate 5-FU resistance and higher risk for distant metastases [20,22,24]. 

This phenotype was validated to be highly predictive of 5-FU chemotherapy response in patients with stage II/III aggressive colon adenocarcinomas [26,54,55,56,57,58,59], but not for patients with rectal cancer treated with 5-FU and levamisole [51,56]. In line with these data, Hestetun et al. reported an unusual high rate of maspin nuclear positivity, in almost all carcinomas of the colon (Table 1) and considered nuclear maspin stain as an indicator of resistance to 5-FU/levamisole regimen [59].

As some of the MSI-H carcinomas might respond to 5-FU, it was suggested that, as cytoplasmic predominance is the most maspin phenotype of these cases, presence of simultaneous maspin/p53 nuclear positivity might be used to select the MSI-H carcinomas with potential 5-FU benefits [24]. 

In high-staged rectal cancers, weak cytoplasmic expression was correlated with positive response to neoadjuvant concurrent chemoradiotherapy (CCRT) but nuclear maspin proved to rather indicate therapeutic resistance [57,59].

### 7.2. Maspin and Anti-Epidermal Growth Factor Receptor (Anti-EGFR) Therapy

For patients with metastatic CRCs who display *EGFR* but not *KRAS* mutations, in the exon 2 (codons 12 or 13), it is recommended to use anti-EGFR monoclonal antibodies (mAb) such cetuximab or panitumumab [24,59]. The newest data showed that up to 20% of patients with CRC who are considered wild-type (for exon 2) might develop *KRAS* mutations in the exons 3 or 4, *NRAS* mutations in the exons 2–4 or point mutations such those described in only one report and refers to *HRAS* mutations (e.g., c.38G>A; p.G13D of the exon 2) [58]. These cases also showed resistance to anti-EGFR drugs [60].

The molecular mechanism of *HRAS* gene-mediated resistance to anti-EGFR mAB was not yet elucidated [60,61]. It might be induced by HER2 and MET genes amplification or by MAPK activation [12,61,62]. As activation of the *EGFR* signaling pathway stimulates maspin phosphorylation with further nuclear translocation [12,61], it might be concluded that nuclear maspin might be an indicator of resistance to anti-EGFR therapy. This fact was hypothesized based on a pre-experimental study using cell lines from non-small cell lung carcinomas [61] and was not yet studied in CRC samples. 

### 7.3. Maspin and Anti-Angiogenic Targeted Therapy

In metastatic CRCs, anti-angiogenic/anti-VEGF-A drugs namely bevacizumab, aflibercept, regorafenib or ramucirumab show promising results in clinical practice, when added to standard therapy [60,63]. As they also affect the preexisting normal mature vessels, the side effects are frequent, and these drugs cannot be used in any patient.

In experimental studies, intravascular administration of adenovirus-maspin proved to exert an antiangiogenic effect against tumor neoformed vessels and endothelial cell apoptosis but did not affect the preexisting normal mature vessels, even after long exposure of mice with CRC [10]. Apoptosis of the neovessels was linked to the apoptotic Bcl-2 gene [7,10]. 

In tumor-related hypoxic medium, obtained in breast cancer cell lines, acetylsalicylic acid (aspirin) proved to increase the level of nitric oxide (NO) and subsequently increase the intracellular and serum level of maspin. This process was p53-dependent and induced a decreasing metastatic potential of tumor cells [7]. We did not find any data to confirm or infirm these aspects in CRC. 

As tumor lymphangiogenesis via maspin seems to be rather modulated by VEGF-C and its receptors VEGF-R2 and VEGF-R3 [14] and by the Hipoxia-inducing factor (HIF-1α) [62], while systemic metastases mainly occur via VEGF-A [63,64], the maspin-based tumor therapeutics should target these specific molecular endpoints. Moreover, due to the existing link between Wnt signaling pathway, ARSB, maspin and angiogenesis [36,65], a simultaneous inhibition of angiogenesis, tumor cell metabolism and EMT might be obtained through maspin modulation targeted therapy [37]. 

### 7.4. Maspin and Immune Checkpoint Blockade

In a subset of CRCs, especially MSI subtype, maspin was experimentally proved to be a tumor associated antigen with immunogenic properties [4,28,66]. Tanaka et al. suggested that maspin might exert a synergistic role with immune checkpoint inhibitors [4]. Dzinic et al. also considered maspin as a modulator of host immune response but the exact mechanism is unknown [66]. The immunomodulatory function of maspin consists on inhibition of the macrophage phagocytosis and stimulation of the inflammatory cytokines production [29] or can be related on T-cell mediated immune response [15]. It does not know why the inflammatory cells are maspin negative and if maspin is, indeed, involved in tumor immunomodulation. It is tempting to believe that targeting maspin might be a novel therapeutic strategy for triggered individualized immunotherapy [28,66]. 

## 8. Concluding Remarks

The above-mentioned aspects indicate that maspin might be a valuable IHC biomarker to help pathologists in their daily practice and improve inter-observer agreement. Adding maspin for daily quantification of buds might help to easily identify high-budding CRCs, particularly those diagnosed in early stages. 

In MSS cases, nuclear maspin might be considered an indicator of high budding degree and high aggressivity but also of possible response to 5-FU-based therapy. As regarding MSI-H cases, if cytoplasmic maspin prove to mark the serrated carcinomas, nuclear positivity might be, indeed, an indicator of a better prognosis.

Large cohort analyses need to be performed to elucidate the two original hypotheses highlighted in the present review, based on literature data and clinical experience of the authors. 

First hypothesis refers to the possible role of maspin in the identification of the CRC tumor cells “at the point of budding”, with possible prognostic or predictive value. The second hypothesis highlights the possible role of maspin in dysregulating the intestinal microbiota and inducing IBD-related CRC. The possible use of maspin-based therapeutics in targeted therapy of CRC should be considered an option for a safer anti-angiogenic therapy with immune blockade synergistic effect.

## Figures and Tables

**Table 1:** Maspin subcellular expression in colorectal carcinomas (CRCs), reflected by the recently published studies that included at least 100 cases.

Authors, Year [Reference]	No. of Carcinomas and Selection Criteria	Cytoplasmic Expression	Nuclear Expression	Prognostic/Predictive Value of Maspin
Boltze et al., 2005 [53]	n = 280 (colon); stages I-IV—TMA blocks-0.6 mm cores	69% of the cases—cytoplasmic or nuclear; loss of expression—left-sided high-grade (G3) metastatic tumors	Not reported	Cytoplasmic overexpression—suppressive role—better prognosis; loss of expression—short OS
Bettstetter et al., 2005 [35]	n = 200 (colon and rectum); 41 MSI and 159 MSS (TMA blocks-2 mm)	72% in MSS/MSI-L and 78% in MSI-H, upregulated in well-differentiated carcinomas (G1)	50% in MSS/MSI-L and 73% in MSI-H; upregulated in high-grade (G3) carcinomas, especially in the invasion front	Cytoplasmic expression—tumor suppression role; Nuclear positivity—tumor progression
Dietmaier et al., 2006 [58]	n = 172 (colon); stage III—before and after 5-FU based chemotherapy	71.5%—no correlation with any of the examined parameters	52.3%—Upregulated in MSI vs. MSS cases and in high-grade (G3) carcinomas	Nuclear overexpression in MSS cases—independent adverse prognostic factor for OS but highly predictive of 5-FU chemotherapy
Umekita et al., 2006 [40]	n = 104 (colon and rectum); stages I–III (whole section)	66% of the cases-correlation with high tumor stage and high budding degree (>9 foci in a field)	Not reported	Cytoplasmic expression—aggressive phenotype but not indicator of OS
Markl et al., 2010 [27]	n = 156 (colon and rectum); stages I/II (TMA blocks-2 mm)	Correlation with high tumor grade (72% of the cases)	Correlation with high tumor budding (48% of the cases)	Cytoplasmic expression —tumor suppression role; For pT3/stage II cases, pure nuclear expression—worse OS; even worst OS in maspin negative cases
Fung et al., 2010 [39]	n = 450 (colon); stage III (TMA blocks-1 mm)	80%—Correlation with right-sided high-grade carcinomas	79%—Correlation with right-sided high-grade carcinomas with at least 4 metastatic lymph nodes	Not independent prognostic value
Hestetun et al., 2010 [59]	n = 380 (colon and rectum); stages II/III (TMA blocks)—before and after chemotherapy	23% of the cases—without other details	99%—in colon cancers, upregulation after chemotherapy	For colon cancer, nuclear overexpression—resistance to 5-FU/Lev chemotherapy and, after chemotherapy, low DFS and CSS; no prognostic or predictive value for rectal carcinomas
Pasz-Walczak et al., 2010 [23]	n = 102 (colon and rectum); stages I–IV (whole section)	88% of the cases—correlated with high tumor grade (G3), advanced stage, presence of metastases	58.82%—no correlation with any of the examined parameters	Cytoplasmic overexpression—poor prognosis
Gurzu et al., 2013 [16]	n = 121 (colon and rectum); 43 stages I/II and 78 stages III/IV (whole section)	44%—cytoplasmic predominance; associated with low budding degree (<5 foci in a field) and p53 negativity, more frequent in distal colon	24%—nuclear predominance and 23% with associated cytoplasmic positivity—mixed expression (40% in MSI cases); nuclear predominance—p53 positive highly angiogenic tumors	Cytoplasmic or mixed expression—better prognosis; nuclear predominance or loss of positivity—low OS; mixed expression in MSI cases—better prognosis;
Baek et al., 2014 [9]	n = 377 (colon); 147 stage I/II and 230 stage III/IV	Correlated with high tumor grade (G3), advanced stage, high budding degree, and lymph node metastases, especially if associates nuclear positivity	Correlation with cytoplasmic expression and more expressed on the right-sided colon cancer	Overexpression—reduced DFS and OS, in correlation with CEA serum level
Snoeren et al., 2015 [26]	n = 419 (colon and rectum): 243 stage II and 166 stage III (TMA blocks-0.6 mm)—before and after chemotherapy	Correlated with right-sided location, high tumor grade (G3), mucinous differentiation and MSI status, especially if associates nuclear positivity	0.95%	Mixed overexpression—independent predictor of recurrence, lymphatic spread and DFS in stages III and IV but not stage II
Kim et al., 2015 [46]	n = 216 MSI-H carcinomas: 139 stages I/II and 77 stage III/IV (TMA blocks-2 mm)	Not reported	51%—associated with CIMP-H status, MLH-1 methylation, advanced stage, metastatic status, high tumor budding	Nuclear overexpression—worse DFS but not independent prognostic value
Tanaka et al., 2020 [4]	n = 743 (colon); 628 stages I/II and 115 stages III/IV (TMA blocks-2 mm)	Correlation with advanced stage and MSI status (16.4% and 57.4% in early vs. late stages; 22.3% vs. 13.9% in MSI vs. MSS carcinomas)	Correlation with MSI status (22.1% vs. 22.6% in early vs. late stages; 36.9% vs. 19.3% in MSI vs. MSS carcinomas)	Overexpression in MSI early-staged CRCs—better prognosis
Banias et al., 2020 [2]	n = 112 (colon and rectum); stages I–III (whole section); 100/112 cases were MSS	52.7% in tumor core and 19.6% in buds—more frequent in low-grade budding non-metastatic epithelial-type carcinomas with LNR ≤ 0.15	27.7% in tumor core and 60.8% in buds—associated with high-grade budding, lymph node metastases, mesenchymal-type carcinomas	Cytoplasmic expression—better OS but not independent prognostic value

CEA—carcinoembryonic antigen; CIMP-CpG—island methylator phenotype; CIMP-H—CIMP-high; CSS—cancer specific survival; DFS—disease free survival; 5-FU-5-fluorouracil; G—grade of histological differentiation; Lev—levamisole; LNR—lymph node ratio; MSI—microsatellite instability; MSI-H—high grade MSI; MSI-L—low grade MSI; MSS—microsatellite stable-tumors; OS—overall survival; TMA—tissue microarray.

## Data Availability

Not applicable
